# Evaluation of the capability and reproducibility of RECIST 1.1. measurements by technologists in breast cancer follow-up: a pilot study

**DOI:** 10.1038/s41598-023-36315-w

**Published:** 2023-06-05

**Authors:** Pierrick Gouel, Françoise Callonnec, Émilie Levêque, Céline Valet, Axelle Blôt, Clémence Cuvelier, Sonia Saï, Lucie Saunier, Louis-Ferdinand Pepin, Sébastien Hapdey, Julie Libraire, Pierre Vera, Benjamin Viard

**Affiliations:** 1 Department of Medical Imaging, Henri Becquerel Cancer Center, Rouen, Normandy France; 2 Department of Statistics and Clinical Research Unit, Henri Becquerel Cancer Center, Rouen, Normandy France; 3 QuantIF-LITIS EA4108, University of Rouen, Rouen, Normandy France

**Keywords:** Breast cancer, Cancer imaging

## Abstract

The evaluation of tumor follow-up according to RECIST 1.1 has become essential in clinical practice given its role in therapeutic decision making. At the same time, radiologists are facing an increase in activity while facing a shortage. Radiographic technologists could contribute to the follow-up of these measures, but no studies have evaluated their ability to perform them. Ninety breast cancer patients were performed three CT follow-ups between September 2017 and August 2021. 270 follow-up treatment CT scans were analyzed including 445 target lesions. The rate of agreement of classifications RECIST 1.1 between five technologists and radiologists yielded moderate (k value between 0.47 and 0.52) and substantial (k value = 0.62 and k = 0.67) agreement values. 112 CT were classified as progressive disease (PD) by the radiologists, and 414 new lesions were identified. The analysis showed a percentage of strict agreement of progressive disease classification between reader-technologists and radiologists ranging from substantial to almost perfect agreement (range 73–97%). Analysis of intra-observer agreement was strong at almost perfect (k > 0.78) for 3 technologists. These results are encouraging regarding the ability of selected technologists to perform measurements according to RECIST 1.1 criteria by CT scan with good identification of disease progression.

## Introduction

The radiological evaluation of the follow-up of solid tumors has based on the RECIST 1.1^1,2^. The evaluation of these criteria is primarily focuses on the identification of the target lesions on CT data and follow-up over time. There were four categories: complete response (CR), partial response (PR), progressive disease (PD), or stable disease (SD). Among these categories, PD determination is particularly important because "progression-free survival" is a significant endpoint, as is "overall survival" as the primary endpoint in clinical trials^3^.

For several years, there has been a shortage of radiologists in many countries^4–6^ while at the same time cancer incidence rates remain high^7–9^ in the world. Consequently, radiology procedures are constantly increasing^10^.

In this context, various organizational strategies have been developed. The first concerns teleradiology, which consists of remote consultation and the interpretation of medical imaging examinations. This telediagnosis solution has developed considerably in recent years, allowing access to care, reinforcing access to qualified radiologists, and providing specific expertise to compensate for the shortage of radiologists in certain hospitals^11,12^. Teleradiology can increase the efficiency of medical imaging; however, its optimal practice is hampered by technological limitations and regulatory impediments^12^ limiting access to the patient's complete medical history and important information to complete the radiological evaluation.

The second approach relies on artificial intelligence (AI), which allows the implementation of a set of computer techniques and theories aimed at simulating or reproducing certain attributes of human intelligence, such as learning or decision-making. AI is very promising in medical imaging^13,14^ and is widely used to predict the evolution of treatment and define the most appropriate treatment. However, it has not yet proven its worth in terms of diagnosis^15^ and prognosis^13,16,17^, and many limitations hinder the prospects for rapid development^13,17,18^.

Lastly, radiography technologists or radiographers could contribute to RECIST 1.1 measurements. The modalities of their professional practice are regulated by a decree specific to each country, and to our knowledge, none authorizes them to participate in the review of imaging examinations. However, in several countries, radiographers are included in the analysis of the examinations performed. In the Netherlands, they are trained to evaluate mammograms to detect possible abnormalities (pre-reading)^19^ with many benefits, such as providing an alert signal to the radiologist^20^.

In this study, we propose to evaluate the classification of RECIST 1.1 measurements performed by technologists on post-therapeutic CT in the follow-up of breast cancer patients in which target lesions were identified by a radiologist on baseline CT.

## Results

### Characteristics of the database

Table 1 details the characteristics of the 90 participants with breast cancer on histology types and tumor classification at the time of their baseline examination. More than 33% had lymph node involvement and 29% had at least one metastasis. In total, for the analysis, 270 follow-up therapy CT were reviewed by each technologist reader, including 445 target lesions identified by an experienced radiologist with more than 5 years’ experience at the time of the baseline examination, for a cumulative total of 10 radiologists over the time frame of the database collection. Of the 270 CT, the RECIST 1.1 response classification given by the radiologists was five CR, 60 PR, 93 SD, and 112 PD (Table 2).

**Table 1:** Characteristics of the 90 patients enrolled at diagnosis.

	n = 90
Age, mean (± sd)	58 (± 12.8)
(min;max)	(24;86)
Gender, n (%)	
Male	2 (2%)
Female	88 (98%)
Histologic finding, n (%)	
Infiltrating ductal carcinoma (IDC)	81 (90%)
Invasive Lobular Carcinoma (ILC)	3 (3%)
Unknow	6 (7%)
Tumor classification, n (%)	
T1	4 (4%)
T2	17 (19%)
T3	8 (9%)
T4	23 (26%)
Unknow	38 (42%)
Node involvement	
N0	19 (21%)
N1	24 (27%)
N2	3 (3%)
N3	6 (7%)
Unknow	38 (42%)
Metastases involvement	
M0	39 (43%)
M1	26 (29%)
Unknow	25 (28%)
TNM stage, n (%)	
Stage I	2 (2%)
Stage IIA	5 (6%)
Stage IIB	8 (9%)
Stage IIIA	3 (3%)
Stage IIIB	21 (23%)
Stage IV	26 (29%)
Unknow	25 (28%)

**Table 2:** Percentage of strict agreement of the RECIST classifications of each technologist reader for all therapeutic follow-up scans (n = 270).

	Progression disease (PD)	Stable disease (SD)	Partial response (PR)	Complete response (CR)
Number of CT scans (n/270)	112	93	60	5
Technologist Reader 1 (%)	83	80.6	56.7	20
Technologist Reader 2 (%)	97.3	60.2	41.7	0
Technologist Reader 3 (%)	75	69.9	51.7	0
Technologist Reader 4 (%)	73.2	74.2	41.7	20
Technologist Reader 5 (%)	84.8	82.8	66.7	20

### Measurement of differences and discrepancy analyses

**Figure 1:**
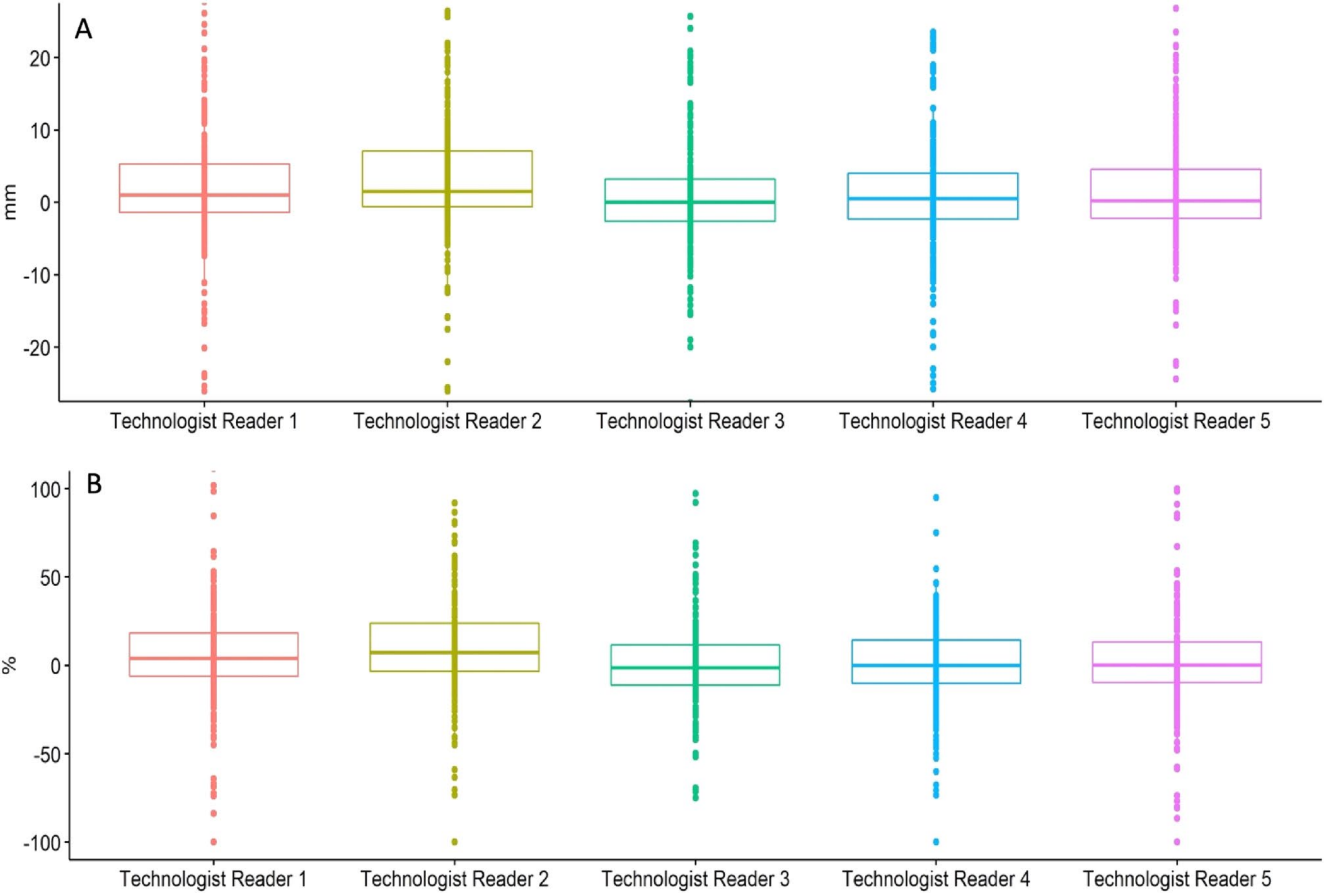
Box plot differences in mm (**A**) and percentage (**B**) of the sum of target lesions between the radiologist and technologist readers. Each point represents the calculated values of all CT reviewed.

Differences in target lesion measurements between technologist readers and radiologists are given in millimeters and percentages, which are calculated as the relative difference with respect to the measurement of the target lesion by the radiologist (Fig. 1) using a box plot. The median was close to 0 for each technologist reader (between − 1.3% and 7.2%) with a low interquartile range but with extreme values.

**Figure 2:**
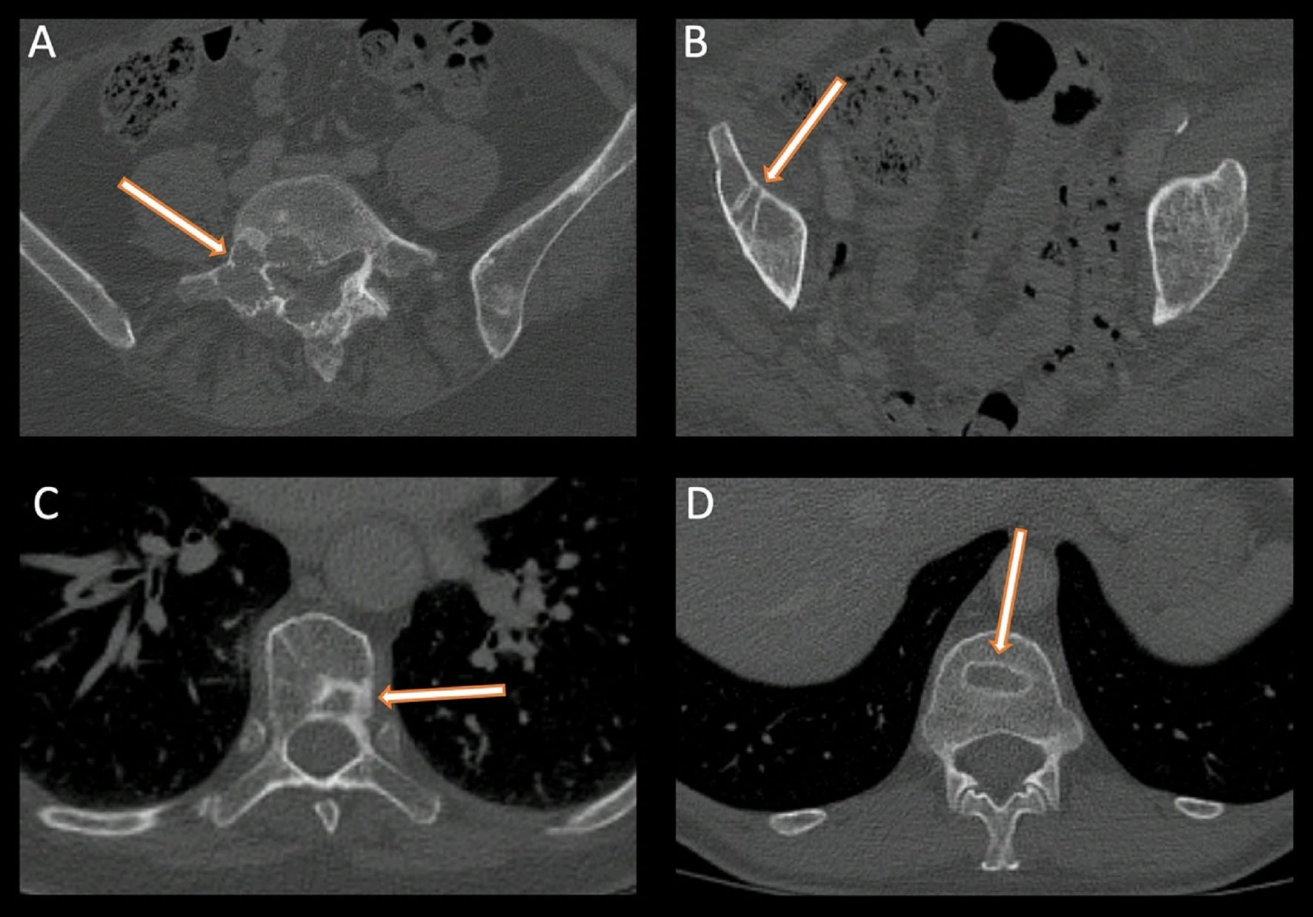
Examples of bone lesions (**A**) responsible for disease progression and bone condensations (**B**–**D**) are interpreted as non-progression. (**A**) is a bone lesion of the lumbar L4-L5 vertebra, (**B**) is a condensation of a lytic lesion of the right iliac wing, (**C**) is a bone condensation of a thoracic vertebra, and (**D**) is a non-pathologic T12 vertebrae compression.

A discrepancy in classification between technologist readers and radiologists that requires review by an independent radiologist (e.g., with at least three technologist readers disagreeing with the radiologists) was found for 102/270 CT. The independent radiologist was in favor of the radiologists in 28/102 (27.5%) of the cases, technologist readers (≥ 3 technologists) in 52/102 (51%) of the cases, and a minority of technologist readers (< 3 technologists) in 22/102 (21.6%) of the cases. Analysis of discordant CT showed mistakes between bone lesions related to the oncological disease and bone condensations of no-oncological causes in 36/102 (35.3%) of cases (Fig. 2), discordance between PR and SD classifications in 22/102 (22%) of cases, and no identification of a lesion in 8/102 (7.5%). In 5.1% (5/102) of the cases, the correct classification was not found by any technologist readers.

### Concordance of RECIST 1.1 classifications

The rate of agreement of the classifications between each technologist reader and that of experienced radiologists for the entire patient population and CT is shown in Table 3. For all classifications according to RECIST 1.1, three technologists obtained moderate agreement values with the original report of the radiologist (k between 0.47 and 0.52) and two substantial agreements (k = 0.62 and k = 0.67). Dichotomizing the classifications of progressive and non-progressive disease (CR/PR/SD vs. PD) improved the overall results (k values between 0.52 and 0.69).

**Table 3:** Results of Cohen's kappa classification agreement and by the percentage of strict agreement of each technologist reader for all therapeutic follow-up scans (n = 270).

	Cohen’s Kappa All classifications	Percentage of strict agreement All classifications	Cohen's Kappa CR/PR/SD vs PD	Percentage of strict agreement CR/PR/SD vs PD
Technologist reader 1	0.621 [0.543;0.7]	75.2	0.674 [0.585;0.763]	84.1
Technologist reader 2	0.525 [0.438;0.613]	70.4	0.539 [0.442;0.637]	75.9
Technologist reader 3	0.496 [0.411;0.581]	66.7	0.524 [0.421;0.627]	76.7
Technologist reader 4	0.473 [0.387;0.56]	65.6	0.606 [0.509;0.704]	81.1
Technologist reader 5	0.675 [0.6;0.75]	78.9	0.69 [0.603;0.777]	84.8

The values in brackets correspond to the confidence interval of Cohen's kappa at 95%.

Radiologists classified a total of 112/270 examinations as PD and 414 new lesions were identified. Individual analysis of each technologist reader by classification (Table 2) showed a percentage of strict agreement of the PD classification between technologist readers and radiologists of substantial to near perfect agreement (range 73–97%).

A total of 168/270 CT showed no more than three discrepancies. The percentage of strict agreement between radiologists was 72% (168 + 28/270). The independent radiologist favored the original radiologist’s report in 28/102 (27.5%) cases. A large number of errors (> 35% of the cases) were related to confusion between a bone condensation considered non-progressive or non-pathological and a bone lesion of cancerous origin often synonymous with PD (Fig. 2).

### Intra-technologist reader concordance

The intra-observer agreement analysis estimated from 30 CT duplicated in the database is presented in Table 4. Technologist readers 3 and 4 obtained moderate reproducibility (k = 0.50 and k = 0.59, respectively), technologist reader 2 (k = 0.78), and technologist readers 1 and 5 showed almost perfect agreement (k = 0.83 and k = 0.89, respectively).

**Table 4:** Intra-technologist readers concordance. The values in brackets correspond to the confidence interval of Cohen's kappa at 95%.

	Cohen’s Kappa
Technologist reader 1	0.832 [0.652;1.012]
Technologist reader 2	0.783 [0.585;0.98]
Technologist reader 3	0.503 [0.253;0.752]
Technologist reader 4	0.593 [0.268;0.917]
Technologist reader 5	0.894 [0.752;1.036]

## Discussion

This pilot study was conducted to evaluate the feasibility of performing RECIST 1.1 pre-reading during CT oncology follow-up in patients with metastatic breast cancer. To our knowledge, this is the first study to determine the ability of technologists to perform RECIST 1.1 measurements and classification.

The differences in mm and percentage measurements achieved by the technologists gave good overall results, with a median close to 0 and a low dispersion, but showed extreme values. These results can be explained by errors in the selection of the lesion or in the choice of CT reconstruction used for the measurement, resulting in a difference in slice thickness and, therefore, in lesion size. Indeed, in our retrospective study, only 25 of the 90 patients underwent a screenshot of the initial measurements performed by radiologists on the e-CRF. This information is crucial for accurately reproducing the measurement of the target lesion, such as the direction and exact position of the one-dimensional segment or even the series used for the measurement. Beaumont et al.^21^ showed a 73% difference in the measurement of the same liver lesion due to a different level of viewing windowing between the two readers.

Our overall results show heterogeneous agreement, depending on the technologist, ranging from moderate to substantial (k = 0.47–0.67) while it is 72% between radiologists (original reports vs. independent radiologists). The agreements improved significantly for technologists when the classifications were dichotomized into progression vs. no-progression (k = 0.52–0.69). Our results are comparable to the results reported by Keil et al.^22^. In this study, with a more favorable design (41 patients, one baseline and one follow-up CT, with three experienced radiologist readers), the RECIST 1.1 classification concordance rate was 76%. In contrast, in a more recent study, Kuhl et al.^23^ showed extremely high agreement (k > 0.97) when readings were performed by three experienced radiologists with the same target lesions identified.

Several studies have shown that the professional experience of radiologists is an important parameter to ensure good classification according to RECIST 1.1^24,25^. Our results are consistent with this, as two technologists with experience > 15 years have higher overall agreement scores than the three technologists with experience < 5 years (K = 0.54 vs. K = 0.49), although experienced technologists have acquired expertise in technical practice, and not necessarily in RECIST 1.1 measurement and classification.

Our sensitivity analysis yielded several promising results. First, the technologists showed a strong ability to identify a CT considered in PD, with percentages of strict agreement ranging from 73 to 97%. This result suggests that technologists should be able to alert radiologists and/or oncologists to possible disease progression.

Skougaard et al.^26^ showed that the number of discrepancies correlated with an increase in the number of reviewers, especially when there were at least three. Fournier et al.^25^ showed that inter-observer variability increased with the number of target lesions identified. In our study, we identified 445 target lesions, which is high compared to similar studies in the literature^27^ and a panel of five technologists.

Our retrospective study has several limitations. RECIST measurements were performed during clinical flow by a radiologist on duty or in teleradiology when the examination was performed. Consequently, several radiologists were involved in the RECIST 1.1 classification reference in our study. This can lead to nonhomogeneous reviews with disagreements in the choice of target and non-target lesions. Therefore, we used independent radiologists who acted as adjudicators when the number of discordances was ≥ 3. In addition, technologists had access only to the strict data needed for the study, which was not the case for radiologist during their practice who had access to the entire patient record, with additional information such as tumor marker results that could potentially guide their interpretation and the RECIST 1.1 classification.

Our study gives encouraging results regarding the ability of some trained technologists to perform measurements according to RECIST 1.1 criteria during CT oncology follow-up with good identification of disease progression and excellent intra-observer reproducibility. Our results complement the study of Beaumont et al.^28^, who showed in a cohort of 23 patients that the involvement of a specialized technician with specific computer tools required few corrections by radiologists (13%) and saved significant time for radiologists.

In the current context of medical shortages, we believe that technologists could help radiologists with new missions, such as pre-reading of exams. However, several uncertainties remain. Technologists need to raise their skill level, especially pathology knowledge, in order to be more efficient, as shown by our analyses of discordant CT scan of oncological and no-oncological bone condensations (mistakes in 35% of cases). At the same time, AI technology also seems to be a promising way for improvement in the coming years^25,29^. However, it is not yet sufficiently effective due to difficulties such as obtaining large structured databases so that the learning of algorithms is optimal^18^. Finally, the association of AI tools and technologists could be an interesting perspective to explore in future studies^30^ in order to provide pre-reports to radiologists who will review them in a reduced time.

The results of this pilot study need to be completed in a large-scale prospective multicentric study to complete our results. Meanwhile, it would be interesting to initiate long-term practical training by companionship with radiologists and to reflect on the integration of the technologists into the process of oncological follow-up of the patients within the framework of cooperation protocol. Without forgetting the question of the responsibility of each one, which will have to be clearly defined, as the final medical conclusion must always be that of the physician.

## Methods

### Patients and constitution of the database

After review and approval by the Institutional Review Board of Henri Becquerel Center (Registration number 2101B), ninety patients with breast cancer (Table 1) who were cared between September 2017 to August 2021 for breast cancer were retrospectively included in this study after giving informed consent for their data to be made available for research purposes and after signing a no-objection attestation form in accordance with the Declaration of Helsinki. All methods were performed in accordance with the relevant guidelines and regulations.

An anonymized database was created using the picture archiving communication system (PACS) Telemis-Medical® (version 4.95, Louvain-la-Neuve, Belgium), including the baseline CT and three follow-up CT examinations of each patient. The same complete and standardized acquisition protocol prescribed by the radiologist was used for all CT scans, with and without contrast medium, with identical dynamic post-contrast injection acquisition times between scans, from orbit to mid-thigh, with a slice thickness of no more than 5 mm according to clinical trial recommendations^31^.

All RECIST 1.1 criteria results of all CT, determined by the radiologist at the time of their performance, were reported on an electronic case report form (e-CRF). The following data were included: at a minimum, the identification, anatomic description, and measurements (in mm) of target and non-target lesions from each examination of axial slices with the CT viewing window selected according to the organ explored, as well as the RECIST tumor classification (CR, PR, PD, or SD). Anonymized screenshots of the identified target lesion measurements were provided for 25 of the 90 patients.

### Radiography technologist selection

**Figure 3:**
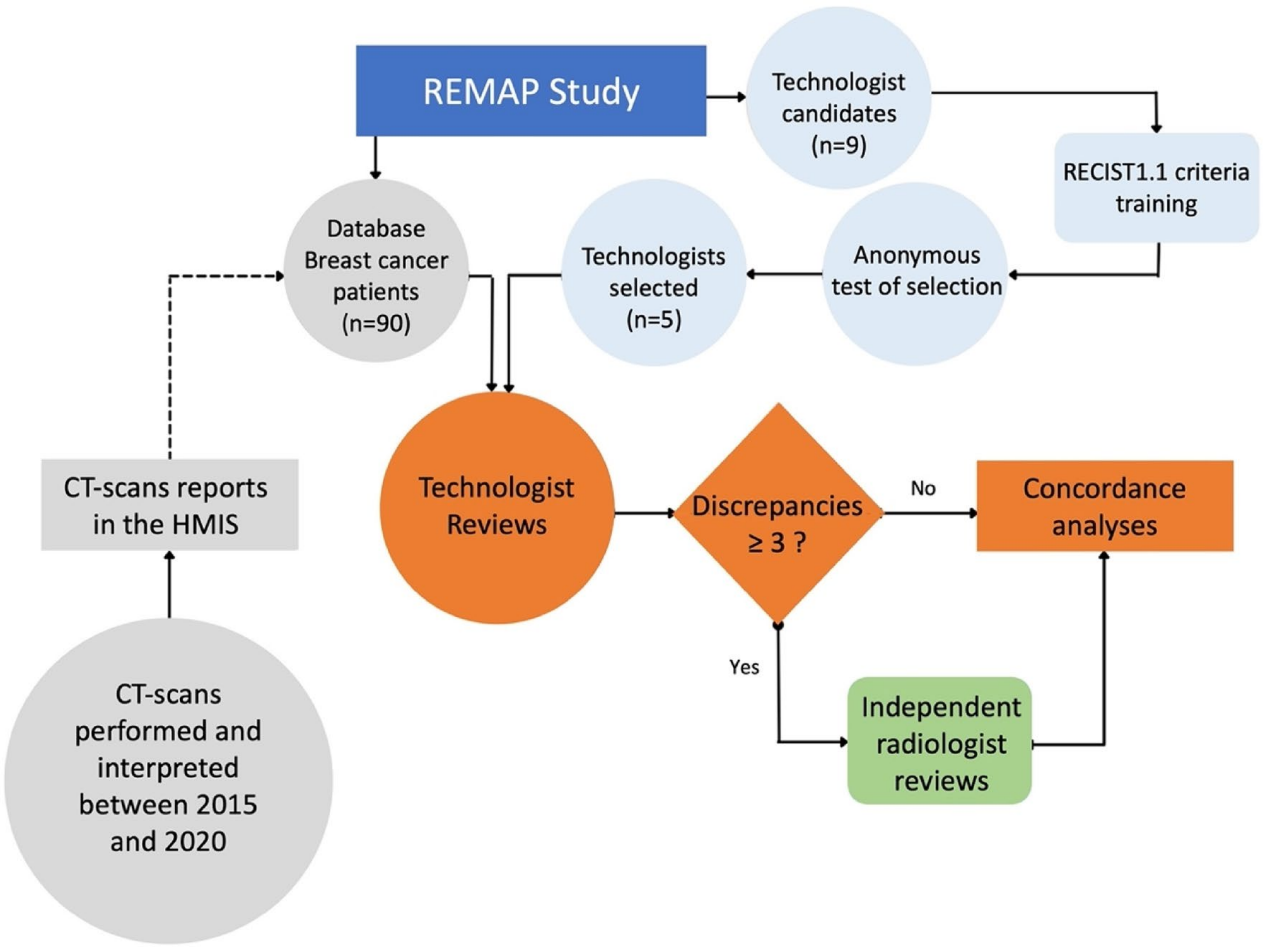
Flowchart of the REMAP study. Independent radiologist reviews were performed when the technologists' RECIST 1.1 classification differed by three or more from the original radiologists' report. HMIS stands for Health Management Information System.

The study was proposed to the whole team technologists working at the oncology CT pole to perform RECIST 1.1 measurements on CT examinations of the study database. Nine technologists were candidates and participated in an anonymous online RECIST 1.1 criteria screening test, identical to that of the radiology residents provided by the French Society of Radiology (SFR), using the Socrative software (version 2.2.5, 1400, 10117 Jasper Avenue NW, Edmonton, Alberta, Canada T5J 1W8)^32^. The five technologists with the best results were selected to participate in the study (Fig. 3). Two selected technologist readers had over 15 years of experience and three had less than 5 years of experience. They then received theoretical training in tumor tracking measures in oncology imaging according to RECIST 1.1 by a radiologist with > 5 years of experience, and e-learning sessions were offered. This training was completed over a period of 6 month by theoretical and practical sessions in real conditions with CTs other than those used in the study as experienced radiologists do to teach radiology residents.

### Response evaluation

The identification number was assigned to five technologist readers who conducted the blinded study. The database was randomized for each technologist reader. To evaluate reproducibility within the review technologist, 10 patients were randomly duplicated in the database without informing the technologist.

To perform RECIST 1.1 measurements on follow-up CT, the technologist reader had access to the lesions identified on the baseline examination by the radiologist, along with the follow-up CT.

In this way, the technologists worked under conditions similar to those of the radiologists when performing lesion measurements for each patient's follow-up. Technologists entered target and non-target lesion dimensions in millimeters in e-CRF and reported the percentage of lesion variations to perform the RECIST 1.1 classification of each patient.

The RECIST 1.1 classification of each technologist's follow-up CT was compared with that of radiologists. When three or more RECIST 1.1 classifications by technologists differed from the original report of the radiologists, an experienced independent radiologist (experience > 5 years) blindly reviewed the corresponding examination. In these cases, the results provided by independent radiologists were then considered as the reference in concordance analyses.

The analysis then focused on the concordance rate of the tumor classification (RC, RP, PD, or SD) of each CT examination. Finally, we analyzed the percentage variation in the sum of the radiologist's lesion measurements with the technologists' measurements.

### Statistical analysis

Characteristics of the patients were described as mean (± standard error) and their extreme values for quantitative variables, and the number and percentage were used to describe qualitative variables.

The difference in the measurement of the target lesion between each technologist reader and radiologist was calculated as the absolute difference in mm and the relative difference in percentage with respect to the radiologist.

The percentage of strict agreement for each classification of RECIST (PD, SD, PR, and CR) was calculated for each technologist reader with respect to the classification of RECIST by the radiologist.

The concordance between each technologist reader and radiologist was evaluated using a Cohen’s kappa, presented with its corresponding 95% confidence interval (CI) for more precision. The following standards^33^ for the strength of agreement for the kappa coefficient were used: ≤ 0, poor; 0.01–0.20, slight; 0.21–0.40, perfect; 0.41–0.60, moderate; 0.61–0.80, substantial; and 0.81–1, near perfect.

The percentage of strict agreement between each technologist and radiologist was also calculated. We also evaluated concordance by grouping the RECIST classifications: CR/PR/SD vs. PD.

For intra-technologist reader reproducibility, 10 patients (corresponding to 30 CT) were blindly evaluated twice by each technologist reader, and we calculated Cohen’s kappa and their associated 95% CI.

## Data Availability

The datasets used and/or analysed during the current study available from the corresponding author on reasonable request.
